# The Complement System, T Cell Response, and Cytokine Shift in Normotensive versus Pre-Eclamptic and Lupus Pregnancy

**DOI:** 10.3390/jcm10245722

**Published:** 2021-12-07

**Authors:** Eugen Ancuța, Radu Zamfir, Gabriel Martinescu, Dragoș Valentin Crauciuc, Codrina Ancuța

**Affiliations:** 1 Research Department, “Elena Doamna” Obstetrics and Gynecology Clinical Hospital, 700398 Iași, Romania; eugen01ro@yahoo.com (E.A.); manager@spitalelenadoamna.ro (G.M.); 2 Fundeni Clinical Institute, 022328 București, Romania; radu.zamfir74@yahoo.com; 3 Department of Internal Medicine, “Grigore T. Popa” University of Medicine and Pharmacy, 700115 Iași, Romania; 4 2nd Rheumatology Department, Clinical Rehabilitation Hospital, 700661 Iași, Romania

**Keywords:** pre-eclampsia, T helper cells bias, complement dysregulation, lupus pregnancy

## Abstract

Successful pregnancy requires an immunological shift with T helper CD4^+^ bias based on disbalance Th1/Th17 versus Th2/T regulatory (Tregs) required to induce tolerance against the semi-allogeneic fetus and placenta and to support fetal growth. Considered a pregnancy-specific hypertensive disorder, pre-eclampsia is characterized by multifaceted organ involvement related to impaired maternal immune tolerance to paternal antigens triggered by hypoxic placental injury as well as excessive local and systemic anti-angiogenic and inflammatory factor synthesis. Both systemic and local Th1/Th2 shift further expands to Th17 cells and their cytokines (IL-17) complemented by suppressive Treg and Th2 cytokines (IL-10, IL-4); alterations in Th17 and Tregs cause hypertension during pregnancy throughout vasoactive factors and endothelial dysfunction, providing an explanatory link between immunological and vascular events in the pathobiology of pre-eclamptic pregnancy. Apart from immunological changes representative of normotensive pregnancy, lupus pregnancy is generally defined by higher serum pro-inflammatory cytokines, lower Th2 polarization, defective and lower number of Tregs, potential blockade of complement inhibitors by anti-phospholipid antibodies, and similar immune alterations to those seen in pre-eclampsia. The current review underpins the immune mechanisms of pre-eclampsia focusing on local (placental) and systemic (maternal) aberrant adaptive and innate immune response versus normotensive pregnancy and pregnancy in systemic autoimmune conditions, particularly lupus.

## 1. Introduction

Overall, pregnancy encompasses multifaceted dynamic physiologic hormonal and immune alterations required to induce maternal immunologic tolerance mechanisms against the semi-allogeneic fetus and placenta and to support fetal growth [1,2,3,4,5]. Beyond profound hormonal modifications (e.g., changes in estrogen and progesterone levels, decreased levels of anti-inflammatory steroids, elevated prolactin, and changes in neuroendocrine axis), successful pregnancy is widely defined by a delicate balance between local and systemic complement activators and regulators as well as an immunological shift comprising T helper CD4^+^ bias (Th1 versus Th2), which is essential to induce maternal immunologic tolerance mechanisms against fetal and paternal antigens [1,2,3,4,5].

Placenta remains a typical fetal-derived organ that borders precisely with the maternal uterine decid the true vascular immunologic tissue interface assisting the coexistence of the semi-allogenic fetus [1,2,6,7,8]. The placental extravillous trophoblast cells invade directly into maternal uterine vessels to enable competent nutrient exchange. In addition, a network of immune cells including macrophages, uterine natural killer cells (NK), dendritic cells (DCs), and also T regulatory cells (Tregs) are widely described in the decidua and are essential for the regular invasion of trophoblast cells during placentation [1,2,3,4,5]. That means that the maternal immune system undergoes unequivocal contact with immunogenic fetal antigens at this maternal–fetal interface. Immune cells interconnect with the invading trophoblasts, practically consent for the remodeling of the uterine spiral arteries, and launch a low resistance system, promoting augmented blood volume and flow to the uteroplacental unit [1,3,4,6,7,8,9]. On the other hand, decidual DCs endorse a local (uterus, placenta) CD4^+^ Th2 bias which is responsible for the maternal immunotolerance to the fetus. Diverse cytokine and angiogenic factors are able to modulate the Th2 shift. Interestingly, successful pregnancy is also related to decreased levels of DCs and NK cells in the peripheral blood [1,3,6,7,8]. Therefore, active immunotolerance is critical to prevent unwanted fetal rejection (pregnancy loss, preterm birth, fetal growth restriction, pre-eclampsia) [1].

Pre-eclampsia (PE) represents a pregnancy-specific hypertensive disorder characterized by multifaceted organ involvement, developing in 4–10% of women in the late phase of gestation [1,2,10,11,12]. Considered a leading cause of maternal and neonatal morbidity and mortality, pre-eclampsia is a heterogeneous obstetric condition clinically defined by new-onset maternal hypertension (systolic blood pressure ≥ 140 mmHg and/or diastolic blood pressure ≥ 90 mmHg) after 20 weeks gestational age, which is usually accompanied by new onset proteinuria (>300 mg in 24 h) or other end-organ damage (liver, brain) [1,10,11,13,14].

Genetic, immunologic, angiogenic, and maternal/environmental factors emphasize the complex pathobiology of pre-eclampsia, triggering placental syncytiotrophoblast stress and ischemia associated with immune activation and systemic vascular response [1,2,6,7,8,9,15]. The disease is widely defined as defective placentation, placental ischemia, abnormal spiral artery remodeling, oxidative stress at the maternal–fetal interface, as well as angiogenic imbalance in the maternal circulation endorsing endothelial and end-organ damage [1,11,15]. The hallmark for pre-eclamptic pregnancies is maternal endothelial dysfunction due to circulating factors of fetal origins, which is responsible for many clinical features such as hypertension, proteinuria, end-organ damage, and intrauterine growth restriction [1,6,7].

Impaired maternal immune tolerance to paternal antigens related to hypoxic injury to the placenta is associated with excessive local and systemic anti-angiogenic and inflammatory factor synthesis promoting a serious, life-threatening vascular complication of pregnancy [1,2,6,7,15]. Local (placental) as well as systemic (maternal circulation) immune abnormalities are widely described in the preclinical pre-eclampsia, comprising aberrant excessive complement activation and impaired adaptive T cell tolerance with an imbalance between Th1–Th17 versus Th2–T regs axis shadowed by increased pro-inflammatory (IL-1, IL-6, IL-17, TNF-α) versus decreased anti-inflammatory (IL-4 and IL-10) serum cytokines [1,2,8,10]. Although the pre-eclamptic pregnancy becomes clinically symptomatic only after 20 weeks of gestation, subclinical disease is hypothesized to initiate at early implantation. The hypothesis was recently validated, as significant alterations in the dynamics of maternal immune system adaptations were depicted months before the clinical onset, and immune system-wide dynamics are disrupted in pre-eclampsia [1,2,9]. Interestingly, although differential dynamics of the maternal immune system were demonstrated in pre-eclamptic versus normotensive pregnancy [1,2,9], it seems that immunological changes described in pre-eclampsia resemble with multifaceted immune abnormalities described in several systemic autoimmune rheumatic conditions such as lupus [1,2,9].

Systemic lupus erythematosus (SLE) is an inflammatory autoimmune multisystem disease underpinned by unpredictable evolution (relapsing–remitting, prolonged remission, persistently active disease), immunologic, genetic and therapeutic heterogeneity, high morbidity, and mortality [16,17,18,19]. As the prototype of autoimmune conditions, systemic lupus remains a multifaceted and multifactorial disease resulting from a complex interplay between environmental factors, hormonal status, as well as overwhelming immunological abnormalities acting on a specific genetic background (polygenic disease). Aberrant immune regulation comprises the activation of both innate and adaptive immunity with impaired clearance of self-nucleic acids related to the induction of type I interferon, lymphocyte activation and signaling, defective T regulatory processes, autoantibody synthesis, complex immune formation and deposition, and multivisceral tissue damage [16,17,18,19,20,21,22]. Several pathways are actually proposed to explain the aberrant immunity in SLE, focusing on the imbalance between production and clearance of apoptotic debris, activated innate and adaptive immunity, inflammatory cell recruitment and tissue injury mediated by the augmented synthesis of pro-inflammatory cytokines derived from activated macrophages (TNFα, IL-6, IL-8), T cells (IL-17) and B cells (IL-10 and IL-6 as a result of IL-21 costimulation), loss of T and B cell tolerance and, finally, excessive autoantibody synthesis partially dependent on a specific type I IFN signature [16,17,18,19]. Extreme patient heterogeneity as well as lupus endotypes rely on immunological heterogeneity comprising the activation of different cell subtypes (B and T cells, plasmablasts, NK cells, neutrophils, and myeloid cells), type I interferon response, and aberrant cytokine profile [16,17,18,19,20,21,22].

Lupus is an immune-mediated disorder characterized by potent dysregulated complement activity, which is associated with a higher risk of obstetric complications, including placental disease (pre-eclampsia, fetal growth restriction, hazard premature delivery), congenital heart block in neonatal lupus, and recurrent miscarriages [16,17,18]. Pregnant women with lupus should be considered at risk due to higher maternal and fetal morbidity, pre-eclampsia, and disease flares, even though the rates of poor maternal and fetal outcomes progressively decreased over the last years [16,17,18]. Hence, lupus pregnancy should always be considered an authentic candidate for an enhanced hazard of premature delivery and intrauterine growth restriction irrespective of disease activity, while the chance for pre-eclampsia remains elevated in those with active disease [16,17,18].

The current review emphasizes the immune mechanisms of pre-eclampsia versus normotensive pregnancy and pregnancy in systemic autoimmune rheumatic conditions such as lupus focusing on local (placental) and systemic (maternal) complement, T cell response, and cytokine shift.

## 2. Overview of Immune Mechanisms in Normotensive Pregnancy

### 2.1. The Complement System in Healthy Pregnancy

Part of the innate immune response, the complement system, involves a comprehensive list of circulating plasma proteins as well as cell-bound components that are generally synthetized by the liver and stimulated during proteolytic cascade irrespective of the activation cascade. It stimulates several processes such as potent inflammatory responses, direct cellular damage through the terminal membrane attack complex (MAC), or pathogens phagocytosis; although the complement system is in the first line and is essential for the defense against pathogens in both circulation and tissues, unwarranted, aberrant activation is responsible for different autoimmune conditions as well as several pregnancy complications such as pre-eclampsia or HELLP syndrome [1,2,9,20,23,24,25,26,27].

**Figure 1:**
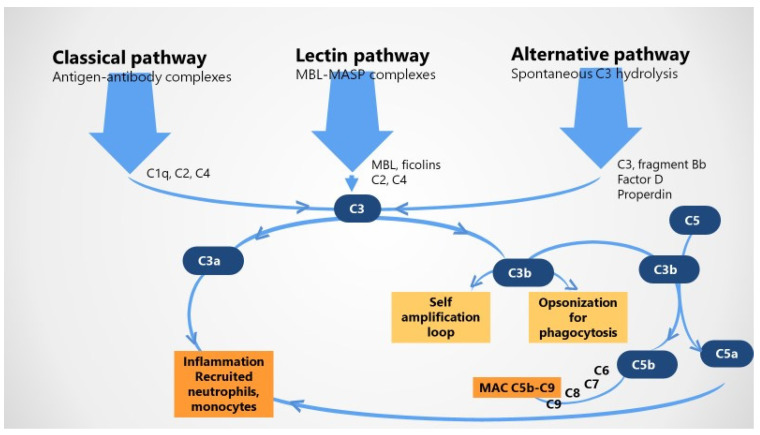
Extracellular complement activation pathways.

Three extracellular complement initiation pathways are actually recognized, all leading to the activation of C3 and of the terminal pathway by the means of C3/C5 convertases [1,8,9,23,24,25], as follows: (i) *the classical pathway* refers to antigen–antibody immune complex-mediated activation of complement, which is recognized by C1q in complex with initially inactive serine proteases C1r and C1s; once both serine proteases are activated, the zymogenic cascade is prompted and cleaves C4 and C2 fractions to form classical pathway C3-convertase C4b2a; (ii) *the alternative pathway* is thought of as an amplification loop and is related to the spontaneous unremitting augmentation of the immune system in response to tissue damage; C3b directly binds to factor B to form C3bB, while factor B and factor D participate to form alternative pathway C3-convertase C3bBb, which can be stabilized by properdin; and (iii) *the lectin pathway* that indicates complement activation mainly related to carbohydrates in the cell walls of microorganisms, especially by mannose-binding lectin (MBL) or one of the 1, 2, 3 ficolins; the MBL-associated serin proteases are able to cleave C4 and C2, generating the C3-convertase C4b2a [1,2,8,20,23,24,25,26,27] (Figure 1).

Irrespective of the activation mechanism, once initiated, the cascade of complement results in activation of the C3 convertase, which releases potent C3a and C3b fractions as a primary step. In the second step, C3a and C5a cleavage products known as potent anaphylatoxins with chemoattractant properties operate as effective pro-inflammatory mediators recruiting neutrophils and monocytes and resulting in local inflammation. In addition, C3b combines with the C5 convertase, cleaves C5, and allows C5b to combine with C6, C7, C8, and C9 to create the terminal MAC C5b–C9 and cell lysis; also, C3b may be engaged in a self-amplification loop and opsonization before phagocytosis [1,2,8,20,23,24,25,26,27].

Beyond its role in the initiation of classical complement cascade, C1q is further involved in immune system modulation, displays antiproliferative effects on T and other peripheral blood cells, intervenes in angiogenesis and clearance of apoptotic cells, and may be critical to the tolerance of peripheral antigens [24,25].

Apart from the extracellular activation, an *intracellular or “complosome” complement activation* pathway was described in TCD4^+^ cells and many non-immune cells; active players include intracellular C3, C3a receptor (C3aR), cathepsin L (constitutively cleaves C3 into C3a and C3b), C5a receptor (C5aR) resulting in IFNγ, and reactive oxygen species production as well as Th1 cells development [20,24,25].

Conclusive data support the crucial intervention of complement in normal pregnancy, demonstrating its role in the basic organization of placenta at the fetal–maternal interface and vascular remodeling of spiral arteries in maternal decidua mandatory for the typical development of pregnancy [1,2,6,7,20,26,27]. Both local and systemic complement activation are typically described during normotensive pregnancy to assure protection of the fetal–placental unit from pathogens [8]. Indeed, healthy pregnant patients display higher levels of complement degradation products as compared to non-pregnant patients, supporting the importance of heightened complement activation during healthy pregnancy [1,9,20,23]. Increased complement activation is important not only to protect against pathogens but also for beneficial placentation; however, excessive and extensive complement activation can result in rejection of fetal-derived tissues [1,20]. Local complement activation (syncytiotrophoblasts and uterine decidua endothelial cells) in response to extravillous trophoblast cells invading into maternal tissues and syncytiotrophoblast (the placental surface constantly exposed to maternal blood) seems to be a reliable step in normotensive pregnancy [1,20,23,24,25,26,27]. Moreover, different studies suggest the role of C1q in the physiological remodeling of decidual spiral artery and partial replacement of endothelial cells by endovascular trophoblasts migrated upward from the anchoring villi. Interestingly, C1q is synthetized in decidual endothelial cells of the spiral arteries, endovascular trophoblasts, but also in the extravillous trophoblasts [1,20,23,24,25,26,27].

On the other hand, complement inhibitors are typically found at the maternal–fetal interface where they are involved in controlling complement activation and protecting the placenta from immunological damage. Membrane-bound regulators including decay-accelerating factor (DAF or CD55), membrane cofactor protein (MCP or CD46), and CD59 are expressed directly on syncytiotrophoblasts and able to avoid unwarranted complement activation and placental injury in successful pregnancy [1,2,20,24,25,26,27]. In addition, several endogenous soluble regulators such as C1 inhibitor (C1INH), complement factor H (CFH), C4 binding protein (C4BP), clusterin, and vitronectin are also able to limit complement activation in healthy gestation [1,2,24].

Successful pregnancy is characterized by a balance between complement activator and regulator factors; regulated complement is required for effective fetal development from implantation to placental development, cervical remodeling, myometrial contractility and delivery, while dysregulated complement is generally involved in implantation failure and early miscarriage, placental ischemia and damage, pre-eclampsia, and preterm birth. Therefore, a subtle balance between the two is essential for pregnancy development, as unregulated complement activation is a threat to pregnancy outcomes [24,25].

### 2.2. Adaptive T Cell Tolerance in Healthy Pregnancy

As mentioned above, pregnancy is defined by complex adaptive hormonal and immunological changes, both systemically and locally (at the maternal–fetal interface), required for the tolerance of the fetus which is considered an immunogenic allograft. Immunological changes refer to the Th2 polarization of the immune response that supports T tolerance to the fetus: switching Th1 (pro-inflammatory) to Th2 (anti-inflammatory) favors the immunotolerance of the fetus [1,2,3,4,8].

The modern immunological paradigm of pregnancy allows for the dynamic Th repertoire, supporting the role of two other potent cellular players in the maintenance of maternal CD4^+^ T cell tolerance to fetal/paternal and placental antigens—pro-inflammatory Th17 cells and regulatory T cells, respectively. Therefore, two main cellular axes with different levels of T cell activation and specific cytokine profiles are actually recognized in pregnancy as follows [1,2,3,4,8]: (i) *Th1/Th 17 axis and derived pro-inflammatory cytokines (IL-1, IL-6, TNFα, IFN-γ);* although Th1 cells are upregulated in very early pregnancy to support premature trophoblast invasion into the uterine spiral arteries, a prompt switch from Th1 to Th2 immunity regularly occurs after placentation, endorsing a frank anti-inflammatory status until delivery; derived from pro-inflammatory T helper cells, Th17 cells are potent actors at the maternal–fetal interface, promoting the synthesis of pro-inflammatory IL-17A cytokine; a lower number of and suppressed Th1 and Th17 cells are typically described in healthy pregnancy; and (ii) *Th2/Treg cells axis and derived anti-inflammatory cytokines (IL-4, IL-10);* undoubtedly, Th2 cells suppress Th1 and Th17 activation through IL-4 and IL-10, respectively; a higher number of activated Th2 as well as systemic and local (decidual) Treg FoxP3 positive cells are characteristic for the successful gestation and help regulate the immune response [9,10]; besides, Th2 polarization and regulatory T cells support a more immune-tolerant status, whereas Th1 and Th17 polarization leads to an excessive pro-inflammatory condition [1,2,3,4,8].

As master regulators of pregnancy, Tregs appear as a result of events initiated at conception and lead to the recruitment of peripheral Tregs into the uterine decidua at embryo implantation [1,8]. Tregs are critical for managing inflammation in the transition to an anti-inflammatory decidual environment essential for embryo implantation and the development of normal pregnancy [1,2,8]. Furthermore, Tregs are increased very early in normal pregnancy and reach their highest levels during the second trimester before decreasing back to normal levels [1,2,8].

Treg can be further classified into natural Treg (nTreg) cells generated in the thymus and adaptive Treg (iTreg) cells derived from naive CD4^+^ T cells in the periphery. iTreg cell expansion occurs in healthy pregnancy, suggesting that this may originate from the decidua, where distinct dendritic antigen-presenting cells reside that are especially proficient to cause iTreg cell induction, which is essential for healthy pregnancy. Decidual Tregs facilitate maternal blood vessel adaptation and the transformation of spiral arteries underpinning placental development [1,2,8,28,29,30,31].

T regulatory cells are able to induce a more potent suppression of responses in the adaptive and immune system and control unwanted immune responses through various mechanisms [6,7]. Interestingly, immune tolerance is dependent on the T cell antigen receptor (TCR) repertoire in decidua Treg known to transform from the first to third trimester of pregnancy. Thus, T helper cells change locally and peripherally in healthy pregnancy; normal pregnancy encompasses (1) upregulated differentiation into Th2 and Treg subsets and derived anti-inflammatory cytokines and (2) inhibited differentiation into inflammatory Th1 and Th17 cells as well as resultant pro-inflammatory cytokines [1,2,8,28,29,30,31].

### 2.3. Cytokines in Normotensive Pregnancy

Overall, successful pregnancy is able to promote a systemically and locally enhanced anti-inflammatory cytokine profile (particularly IL-4 and IL-10) interrelated with a decreased pro-inflammatory cytokine pattern (especially TNFα/IFN-γ and IL-17/IL-22). Indeed, inflammatory response via the Th1 activation pathway is inhibited as a consequence of abnormal Th1 count with low levels of resultant TNFα and IFNγ; in addition, low number and activation of Th17 resulting in reduced IL-17 and IL-22 synthesis may also contribute [1,2,4,5].

On the other hand, immune tolerance mechanisms are enhanced based on high number and activation of Th2 cells via increased IL-2 and IL-4 and promote IL-4, IL5, IL-9, and IL-13 production; supplementary, Treg cells are activated by IL-2, IL-10, and TGFβ and induce an increased expression of IL-10 and TGFβ [1,2,3,5].

A closer look at anti-inflammatory cytokine IL-10 emphasizes its key role in pregnancy; IL-10 is produced by Tregs and villous cytotrophoblasts during pregnancy and is able to activate Tregs development derived from naïve T cells. Both IL-10 and its receptor are upregulated on various cells located in the decidua, including trophoblasts, stromal cells, macrophages, and uterine NK cells. It was suggested that IL-10 supports allogenic tolerance of the fetus by influencing the synthesis of Th1 inflammatory cytokines (IL-2, IFNγ, TNFα) and reducing the inflammation at the fetal–maternal interface. In addition, different studies have demonstrated high levels of IL-10 during normal pregnancy and a dramatic decrease during delivery [1,2,5,28,29,30,31].

**Figure 2:**
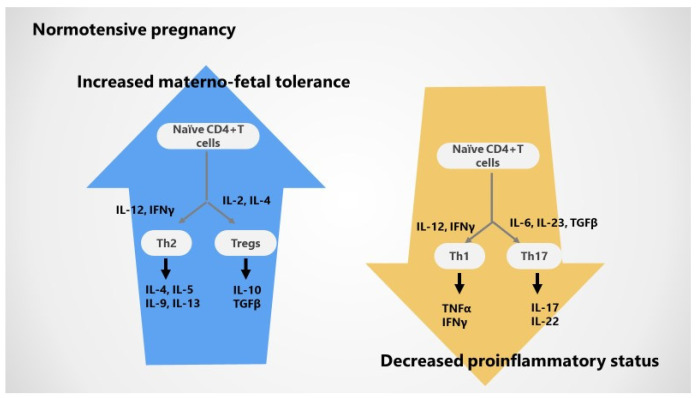
Immunological changes in normal pregnancy; polarized Th2 response with activated Th2–Tregs axis and anti-inflammatory cytokines (IL-4, IL-10) versus inhibited Th1-Th17 cells and their pro-inflammatory cytokines (IL-6, TNFα, IL-1).

Figure 2 emphasizes immunological changes in normal pregnancy.

## 3. Immune Mechanisms in Pre-Eclamptic Pregnancy

Identified as new-onset maternal hypertension, proteinuria, and progressive multi-organ injury associated to pregnancy, pre-eclampsia is a distinct systemic syndrome ranging from asymptomatic and perhaps not diagnosed until delivery cases to early-onset pre-eclampsia with rapidly progressive multi-organ dysfunction, including neurologic (headache, seizure, stroke), hematologic symptoms (thrombocytopenia, disseminated intravascular coagulation), acute renal failure, hepatic (subcapsular hematoma or rupture) disease, even HELLP (hemolysis, elevated liver enzymes, low platelets) syndrome [1,6,7,32,33,34,35]. According to gestational age, pre-eclampsia is classically stratified in two main clinical phenotypes, EOPE or early-onset pre-eclampsia, which is diagnosed before 34 weeks of gestation, and LOPE or late-onset pre-eclampsia, occurring from 34 weeks of gestation [33]. Several risk factors for pre-eclampsia are already recognized comprising major factors (e.g., prior pre-eclampsia, chronic hypertension, pregestational diabetes, multiple gestation, obesity, antiphospholipid syndrome), other risk factors (nulliparity, change in paternity from prior pregnancies, autoimmune maternal disease—lupus, prior placental abruption, assisted reproductive technology, advanced maternal age >35, chronic kidney disease, genetic susceptibility) and rare risk factors (trisomia 21 fetus, family history of pre-eclampsia) [1,2,11,14,36,37]. As there is no treatment to stop disease progression, current management of pre-eclampsia relies on fetal and placental delivery aiming to improve maternal status often at the cost of neonatal outcomes [2,11,14].

Whereas placental dysfunction is associated with anti-angiogenic and inflammatory factors released into maternal circulation, systemic vasoconstriction leads to maternal hypertension and impaired blood supply to major organs such as kidney, brain, and heart, which is correlated with major organ damage. Therefore, dysregulated angiogenesis is an essential event in the pathogenesis of pre-eclampsia [2,6,7,15], promoting a two-stage disease [33]. The first stage, abnormal placentation or placental stage of pre-eclampsia, refers to defective trophoblast invasion of maternal spiral arteries, failed remodulation of maternal spiral uterine arteries, inadequate maternal blood supply, placental ischemia, and hypoxia with final release of trophoblast microparticles into maternal circulation [1,2,23]; these placental microparticles are thought to activate innate and adaptive immune responses, resulting in aberrant complement regulation as well as disturbance in the regulation of the coagulation system, microthrombi formation, endothelial damage, and, finally, failure of the systemic maternal–fetal immunological tolerance [1,2,6,7,23]. The second stage, maternal syndrome or systemic stage, is widely characterized by a systemic endothelial dysfunction leading to vasoconstriction and hypertension, increased inflammatory cytokines synthesis with abnormal excessive inflammation, and increased vascular permeability accompanied by proteinuria and edema [1,2,6,7,23].

A closer look to the complex pathobiology of pre-eclampsia has highlighted the key role of immunological factors (T helper bias) in shallow placentation with reduced placental perfusion and intrauterine growth restriction (pre-eclampsia stage I corresponding to the first and second trimester), overwhelming the maternal–fetal immune tolerance. Pre-eclampsia stage II matching the third trimester is prompted by increased circulating levels of hypoxia-stimulated factors (soluble fms-like tyrosine kinase-1, sFLT1, and soluble endoglin, sEng), decreased circulating levels of placental inhibitor growth factor (PIGF) and vascular endothelial growth factor (VEGF) together with other predisposing factors such as obesity, pro-inflammatory factors, and angiotensin II type-1 receptor autoantibodies (AT1-AA) [1,6,7,23,32,38].

**Figure 3:**
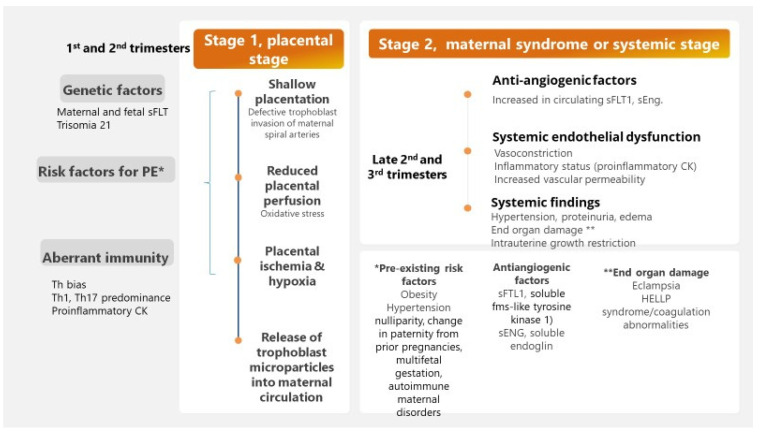
Schematic pathogenesis of pre-eclampsia.

Figure 3 illustrates main events in the pathogenesis of pre-eclampsia.

As already mentioned, the holistic approach of pathogenetic mechanisms of pre-eclampsia should also highlight the role of excessive extracellular and intracellular complement activation as well as impaired T cell tolerance with a dysregulated immune response and a change in T helper cytokines commuted to an imbalance similar to that reported in diverse autoimmune conditions, particularly lupus [1,10,11].

### 3.1. The Complement System and Adverse Pregnancy Outcomes

Beyond its competence in physiologic modifications at the maternal–fetal interface activated in normal pregnancy, complement system dysregulation may promote multifaceted obstetric complications. Aberrant and excessive activation of complement at both the maternal–fetal interface and in the peripheral blood is frequently associated with pregnancy pathology, being a key event that occurs early in the pathobiology of pre-eclampsia and impaired fetal growth [1,2,8,20,24,25,27] being responsible for defective clearance of apoptotic placenta-derived debris in pre-eclamptic pregnancy [1,23].

Interestingly, recent evidence showed that serum levels of complement fractions implicated in the classical activation pathway such as C1q, C4a, and C4b are decreased in both early and late stages of pre-eclampsia, supporting data from experimental models where C1q has a protective role against pre-eclampsia and C1q deficiency associates with endothelial dysfunction, decreased placental vascular endothelial growth factor, and elevated levels of sFlt-1 [19]; thus, C1q and C4 may prevent the onset of pre-eclampsia [1,2,20,39,40,41].

Mannose binding lectin may also contribute to the pathogenesis of the disease, since high levels were reported in women with severe pre-eclampsia [39,40,41].

Several studies have indicated an increase in serum levels of alternative complement activation products; besides, the levels of anaphylatoxins (C3a, C5a) and sC5b–C9 are present in excess in pre-eclampsia compared to normal pregnancy but can also be considered predictors of pre-eclampsia. Additionally, elevated levels of Bb fragment derived through an alternative complement activation pathway may be detected in preclinical pre-eclampsia, representing an early biomarker of pre-eclamptic pregnancies; additionally, Bb fragment was suggested as a predictor of PE [1,2,7,20,24,25,41,42,43,44].

Unwarranted activation of complement at the placental level is directly responsible for placental injury, the release of anti-angiogenic factors, and resultant placental ischemia. Furthermore, increased expression of other split complement products in the syncytiotrophoblast and the degree of C4d, C5a, and MAC deposits in the placental tissue correlated with anti-angiogenic sFms1 in pre-eclampsia [7,43,45].

Conversely, it seems that high expression of the CD55 and CD59 as membrane complement regulators in placental tissues in pre-eclamptic pregnancy represents an attempt to edge local complement activation [17,43,45].

The link between aberrant complement activation and alterations in T cells and their cytokine pattern was recently documented in pre-eclamptic pregnancies; the intracellular complement activation in T cells (“complosome”) represents one important step in the complex pathogenesis of pre-eclampsia. Hence, it seems that C3 and C5 may be activated within intracellular CD4^+^ T cell lysosomes; released from the lysosome, the C3 fraction is cleaved into C3b by lysosomal cathepsin L and shapes the T cell activation through complement receptors on their surface. Intracellular C5 is cleaved into C5a and activates intracellular signaling and Th1 differentiation [1,2,8].

### 3.2. Impaired T Cell Tolerance in Pre-Eclampsia

Pre-eclampsia is classically defined by an increased innate immune activation at the uteroplacental unit and in the periphery. Early activation of CD4^+^ Th1 cells, cytolytic NK cells, and B cells is responsible for the shallow trophoblast invasion and vascular endothelial dysfunction and may result in abnormal maternal vascular remodeling of the placental unit in pre-eclamptic pregnancy. Consequent, augmented uterine artery resistance with reduced placental blood flow and low oxygenation may contribute to placental ischemia and intrauterine growth restriction of the fetus, which are two critical events related to pre-eclampsia [1,2,6,7,10,11]. It was suggested that chronic immune activation with T cells dysregulation together with placental ischemia promotes a pro-inflammatory status orchestrated by high inflammatory cytokines levels unbalanced by decreased anti-inflammatory, regulatory cytokines; furthermore, the bias between pro- and anti-inflammatory events is accentuated during pregnancy, prompting pre-eclampsia to become clinically symptomatic [1,2,8,10,11].

The systemic and local Th1/Th2 shift further expands to Th17 cells and their cytokine profile (IL-17), which are increased and complemented by suppressive Treg and Th2 cytokines (IL-10, IL-4) in pre-eclampsia. In addition, alterations in Th17 and Tregs cause hypertension during pregnancy throughout vasoactive factors and endothelial dysfunction, providing an explanatory link between immunological and vascular events in the pathobiology of pre-eclampsia [1,2,6,7,46,47,48,49,50].

The predominance of pro-inflammatory Th1 cells and Th17 cells further supports a pro-inflammatory, fetal rejection phenotype of pre-eclampsia promoted by the increase in pro-inflammatory T helper cells, and Th17 cells will increase the release of specific pro-inflammatory cytokines, recruitment of neutrophils, and production of oxidative stress in the placenta of pre-eclamptic patients [1,2,6,7,51].

Alternatively, Th2 and Tregs and their anti-inflammatory cytokines are downregulated and further contribute to the dysregulation of immune response during pre-eclamptic pregnancy; also, the proportion of circulating and decidual Treg cells and decidual is declined and associated with defective suppressive function, while Th17 cells are enhanced as well as CD8^+^ effector cells and trophoblast apoptosis [1,2,8,51]. Insufficient and inadequate Tregs priming may promote an altered decidual milieu [8] and a subsequent limited invasion of trophoblasts and maternal vessel remodeling, “shallow” placentation, pre-eclampsia, and/or fetal growth impairment in later gestation [8]. In addition, the decrease in Tregs parallels the severity of the pre-eclampsia clinical scenario [1,2,6,8]. Tregs may also exhibit phenotypic plasticity and lineage instability related to the hyperinflammatory status, with a capacity to shift the phenotype and express cytokines that are characteristic of the T effector subpopulation [8,49]. Interestingly, while abnormal Treg function and number are typically described in autoimmune conditions, during pregnancy, these modifications are clearly related to pregnancy-related pathology, particularly maternal loss of immunotolerance toward the fetal antigens. Thus, Tregs are important players in the pathobiology of pre-eclampsia consenting for an overwhelmed characteristic pro-inflammatory status, as such cells allow the augmented activation of Th1 and Th17 cells as well [1,2,8,31,49]. A closer look to different Tregs subtypes demonstrates that iTregs, known to be actively involved in maternal tolerance for the semi-allogenic fetus, are unreliable in pre-eclampsia; therefore, the impaired induction and development of iTregs remains one important step in chronic peripheral as well as local inflammation in PE. Moreover, biased T cell antigen receptor (TCR) in decidua Treg and reduced clonal expansion at delivery are confirmed in PE women [1,2,8,49].

### 3.3. Cytokines Variations during Pre-Eclampsia

Upregulated Th17 cells via resultant IL-17 are able to mediate hypertension, oxidative stress, and AT1-AA in pre-eclampsia [1,2,6,7,51,52]. Since decreased Tregs and their specific cytokines such as IL-10 and TGFβ are impaired in pre-eclampsia, unrestricted T cells activation and differentiation into a pro-inflammatory phenotype support a pro-inflammatory environment with abundant TNFα, IL-6 and IL-17, advancing endothelial dysfunction, oxidative stress, B cells stimulation with high AT1-AA synthesis, and abnormal blood pressure. Therefore, IL-10 is widely recognized as a key player in pregnancy by neutralizing pro-inflammatory cytokines, AT1-AA, placental oxygen species as well as endothelin-1 [1,2,6,7,49,52]. Conversely, dysregulated IL-10 comprises lower IL-10 levels in pre-eclampsia with further enhanced inflammation and release of anti-angiogenetic factors, irrespective of the severity of pre-eclampsia [1,2,6,7].

**Figure 4:**
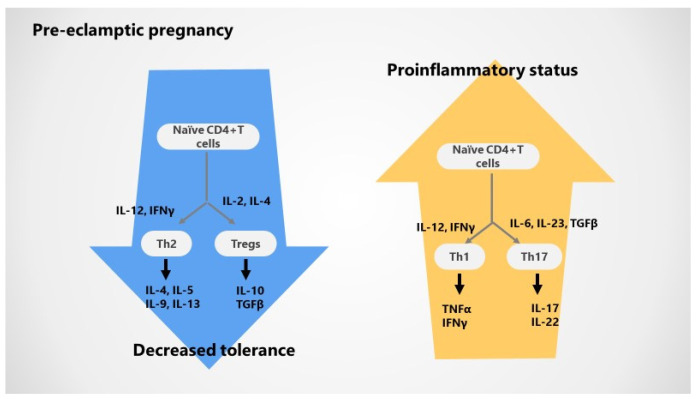
Immunological alterations during pre-eclamptic pregnancy; T-cell bias with Th1–Th17 versus Th2–Tregs axis and resultant cytokines disbalance: decreased maternal–fetal tolerance accompanied by excessive pro-inflammatory status.

Figure 4 shows immunological alterations in pre-eclamptic pregnancy.

An overview of local (maternal–fetal interface) and systemic (peripheral blood) immune mechanisms responsible for pre-eclampsia are summarized below (Table 1).

## 4. Immune Alterations in Lupus Pregnancy with and without Pre-Eclampsia

Diverse studies emphasized risk factors for adverse pregnancy outcomes such as active lupus and particularly high disease activity in the six months before conception, active nephritis at conception or history of nephritis, use of anti-hypertensive medications or proteinuria more than 1 g daily, serological activity, hypocomplementemia during gestation, previous pregnancy complications, glucocorticoid use (prednisone considered a surrogate marker for active disease), and excessive complement activation through an alternative pathway [16,17,18,19,20]. Risk stratification of lupus pregnancy complications classically relies on maternal characteristics (age, smoking, previous pregnancy complications), disease characteristics (activity and organ damage, presence of specific anti-Ro/La and antiphospholipid antibodies, low complement levels) as well as medication (embryotoxic drugs). Thus, pregnancy risks may be classified as low (no lupus activity, absent autoantibodies), intermediate (no activity, present autoantibodies), and high risk (activity, irreversible damage, present autoantibodies) [16,17,18,19].

The main challenge in lupus pregnancy is to promptly diagnose lupus flare, recognizing differences between disease activity from physiological pregnancy changes, to distinguish lupus activity and nephritis from pre-eclampsia [16,17,18,19].

### 4.1. Complement during Lupus Pregnancy

Unwarranted complement activation in lupus patients, particularly through an alternative pathway, as well as low serum levels of circulating complement and its activation fractions are significant biomarkers of disease activity. Complement deposition on immune cells with subsequent organ damage and decreased levels of C1q, C3, and C4 strongly correlates with disease activity and precedes clinically evident flare [1,16,17,18,19,20,21,22]. Despite the well-known dysregulation and variability in complement levels throughout pregnancy, there is a lack of agreement on complement levels as biomarkers in lupus pregnancies [39]; different studies have pointed out correlations between complement and its activation products and adverse pregnancy outcomes as well as specific lupus manifestations, while others failed to demonstrate any association. Therefore, it seems that C3 and C4 levels may or may not be predictive for poor pregnancy outcomes such as preterm pre-eclampsia, spontaneous abortion, premature birth, and stillbirth; likewise, Ba, and Bb fragments and sC5b-9 MAC among lupus pregnancy with positive antiphospholipid antibodies are sensitive indicators of excessive complement activation and predictive for not only for lupus flare but also for pregnancy complications (the PROMISSE study) [1,2,20,21,44,50,53].

In addition, it seems that soluble MAC and alternative pathway products meaningfully correlate with pre-eclampsia in lupus pregnancy with or without antiphospholipid antibody syndrome, depicted early (in the first trimester) particularly in a specific genetic background (heterozygous mutations in CFI, MCP, and CFH genes). Data collected in recent years has demonstrated increased placental complement component deposition, aberrant activation, as well as local inflammation in lupus pregnancy [19,20].

### 4.2. T Cell Responses and Cytokines Variations during Lupus Pregnancy

Apart from immunological changes characteristic for normal gestation, lupus pregnancy is generally characterized by higher serum pro-inflammatory cytokines including IL-17, less Th2 polarization, defective and lower number of Tregs, and the potential blockade of complement inhibitors by anti-phospholipid antibodies. In particular, a significant disbalance between Tregs and Th17 cells was described in lupus and other autoimmune conditions and was associated with immunological dysregulation in pre-eclampsia, emphasizing the potential poor outcomes of lupus pregnancy [1,2,9].

Recent studies focused on altered Tregs and oxidative stress in lupus, since both are involved in immune system dysregulation, abnormal activation and processing of cell-death signals, and autoantibody production [1,19,22]; moreover, while an increase in Tregs and different antioxidant mechanisms to combat oxidative stress are essential for successful pregnancy, decreased Tregs may be associated with a loss of fetal tolerance and a pre-term cessation of pregnancy [1,2,22]. Interestingly, CD8^+^CD25^+^ Tregs appear to be increased in women with lupus in comparison to healthy gestation, while CD4^+^CD25^+^ Tregs are decreased in lupus pregnancy; the explanation relies on an altered IL-2 profile during pregnancy that is able to activate CD8^+^ Tregs more potently than CD4^+^ Tregs, higher levels of CD8^+^ Tregs subpopulation being necessary to maintain pregnancy [19,22]. In addition, lupus pregnancy is associated with high oxidative stress. Since low oxidative stress and altered CD4^+^CD25^+^ Tregs are essential for safe pregnancy, lupus pregnancies are at high risk for potential complications such as pre-eclampsia and miscarriage [19,22].

As above mentioned, pre-eclampsia is widely characterized by poor placentation with disbalance between pro- and anti-angiogenic factors, poor placental perfusion, impaired angiogenesis, increased vascular resistance in the uterine artery, increased complement activation, as well as elevated type I interferon signature; all these alterations are previously described in lupus pregnancy, suggesting similarities between immune alterations in pre-eclampsia and autoimmune conditions as well as pregnancy complications [1,20].

## 5. Conclusions

Successful pregnancy requires an immunological shift comprising T helper CD4^+^ bias (Th1 versus Th2), which is required to induce maternal immunologic tolerance mechanisms against the semi-allogeneic fetus and placenta and to support fetal growth.

Pre-eclampsia remains a severe life-threatening pregnancy complication in both the general population and patients with systemic autoimmune rheumatic conditions such as systemic lupus erythematosus; pre-eclamptic pregnancy is definitely linked to impaired maternal immune tolerance to paternal antigens prompted by defective placentation, hypoxic injury to the placenta, excessive local and systemic complement activation, T cell bias, as well as an augmented pro-inflammatory status.

Systemic autoimmune disorders and particularly lupus are widely characterized by the aberrant activation of both innate and adaptive immunity, inflammatory cell recruitment, tissue injury mediated by the augmented synthesis of pro-inflammatory cytokines, and loss of T and B cell tolerance.

## Figures and Tables

**Table 1:** Immunological alterations in pre-eclampsia.

Immune Pathway	Local/Maternal–Fetal Changes	Systemic Changes/Peripheral Blood
**T helper bias**		
Th1	Increased TNFα	Increased TNFα
Th2	Decreased IL-4	Reduced IL-4
Tregs	Reduced FoxP3 and IL-10 expression in first trimester and at delivery	Decreased Treg proportion and suppressive capacity
	Decreased Treg clonal expansion	Lower activation of memory T reg
Th17	Increased TCD4	Upregulated Th17 cells
**Aberrant complement activation**		
Classical pathway	C1q deposition in chorionic villi, placental blood vessels, endothelia	Low or relative stable C1q
Lectin pathway	Higher C4d, ficolins, H, L deposition in syncytiotrophoblast	Low levels of C4, Ficolins, H
Alternative pathway	C3 deposition in decidua, villous endothelial cells	Higher levels of Bb
Anaphylatoxins (C3a, C5a)	Lower C3a receptor and conflicting results of higher and lower C5a expression	Higher C3aHigher or normal C5a
Terminal MAC (C5b-C9)	Increased MAC deposition in stroma and syncytiotrophoblast	Higher MAC
